# Reappraisal of effects of serum chemerin and adiponectin levels and nutritional status on cardiovascular outcomes in prevalent hemodialysis patients

**DOI:** 10.1038/srep34128

**Published:** 2016-09-26

**Authors:** Hung-Yuan Chen, Yen-Lin Chiu, Shih-Ping Hsu, Mei-Fen Pai, Ju-Yeh Yang, Hon-Yen Wu, Yu-Sen Peng

**Affiliations:** 1 Division of Nephrology, Department of Internal Medicine, Far Eastern Memorial Hospital, New Taipei City, Taiwan; 2 Division of Nephrology, Department of Internal Medicine, National Taiwan University Hospital and National Taiwan University College of Medicine, Taipei, Taiwan

## Abstract

Although chemerin, an adipokine, increases the cardiovascular (CV) risk in obese people, it is associated with a survival advantage in incident hemodialysis (HD) patients. We explored the potential effects of chemerin on CV outcomes in prevalent HD patients. This prospective study included 343 prevalent HD patients. The composite outcome was the occurrence of CV events and death during follow-up. We used multivariate Cox regression analysis to test the predictive power of different chemerin and adiponectin levels and geriatric nutritional risk index (GNRI) for the outcomes. HD patients with higher chemerin levels (≥211.4 ng/mL) had a lower risk of CV events (adjusted hazard ratio [HR], 0.64; 95% confidence interval [CI], 0.41–0.98) and composite CV outcome (adjusted HR, 0.67; 95% CI, 0.45–0.99) than those with lower chemerin levels (<211.4 ng/mL). When evaluating CV outcomes, we identified an interaction between chemerin levels and GNRI, but not between chemerin and adiponectin levels. The findings remained robust in the sensitivity analysis. Thus, in prevalent HD patients with negligible residual renal function, higher chemerin levels predict more favourable CV outcomes.

Chemerin, a novel adipokine, is associated with non-alcoholic fatty liver disease in obese people and in associated with glucose intolerance, dyslipidemia, and visceral adiposity in the general population^1^,^2^. Although chemerin is linked to these critical cardiovascular (CV) risk factors, its association with CV outcomes in the general population remains uncertain^3^,^4^,^5^. In obese children, chemerin induces the expression of intercellular adhesion molecule-1 and E-selectin in endothelial cells *in vitro*^6^. Moreover, in 245 adults with newly diagnosed type 2 diabetes mellitus (DM), chemerin levels were independently associated with endothelial dysfunction and early atherosclerosis, indicated by enhanced carotid intima-media thickness^7^. The evidence indicates that chemerin has a pro-atherogenic effect in people with metabolic problems. However, conflicting reports remain on the effects of chemerin on CV outcomes^3^,^4^,^5^. A study reported a remarkable survival advantage in incident hemodialysis (HD) patients with a residual glomerular filtration rate (GFR) of approximately 7–8 mL/min and higher chemerin levels^8^. Nevertheless, this survival advantage become non-significant after adjusting for residual GFR and chemerin levels are reported to be negatively correlated with GFR^9^. Furthermore, we previously demonstrated a positive association of chemerin and fetuin A levels with nutritional status in patients with prevalent HD^10^. High fetuin A levels and high nutritional status are recognised as predictors of favourable long-term CV outcomes in the HD population^11^,^12^,^13^. Therefore, determining the effect of chemerin levels on CV outcomes in prevalent HD patients is of interest.

Extensive studies have focused on another major adipokine, adiponectin, in HD patients; nevertheless, the effects of adiponectin levels on CV outcomes remain inconclusive^14^,^15^. However, in large-scale studies, an increased risk of CV events, particularly higher risks of sudden death and stroke, have been observed in HD patients with higher baseline adiponectin levels^14^,^15^; moreover, higher adiponectin levels were independently associated with a three-fold higher risk of death in HD patients^16^. Therefore, the roles of adipokines in predicting CV outcomes of HD patients and the relationship between the two crucial adipokines chemerin and adiponectin in HD patients should be carefully reappraised.

This prospective study explored the long-term effects of chemerin on the composite CV outcome and identified its potential interaction with nutritional status in predicting CV outcomes. We also determined the relationship between chemerin and adiponectin and their effect on the CV outcomes of prevalent HD patients with negligible residual GFR.

## Results

### Baseline characteristics of participants

The baseline characteristics of the participants are summarised in Table 1. Compared with those with lower chemerin levels, patients with higher chemerin levels had higher serum albumin, serum calcium, and triglyceride levels and geriatric nutritional risk index (GNRI), but lower adiponectin levels.

### Outcomes

In total, 97 participants experienced CV events, 21 died because of CV events, and 118 presented the composite CV outcome. Of the 97 patients with CV events, 17 had intracranial haemorrhage or ischemic stroke, 56 had coronary artery disease (either non-fatal acute myocardial infarction or coronary revascularization or coronary bypass surgery), 7 had peripheral arterial occlusion disease, and 17 were hospitalised for heart failure. Of the 21 patients with CV death, 8 deaths occurred because of coronary artery disease and 13 had sudden cardiac deaths. The median follow-up period was 2.5 years (range, 0.05–4.2 years).

### Correlation between chemerin, high sensitivity C-reactive protein (hs-CRP), and adiponectin levels and GNRI in prevalent HD patients

Chemerin levels were positively correlated with GNRI (Spearman’s rho = 0.22, *P* = 0.03) and with serum albumin levels (Spearman’s rho = 0.18, *P* = 0.03). However, chemerin levels were not correlated with hs-CRP levels (Spearman’s rho = −0.05, *P* = 0.4) in our patients. Notably, chemerin levels were negatively correlated with adiponectin levels (Spearman’s rho = −0.29, *P* < 0.001). Adiponectin levels were negatively correlated with hs-CRP levels (Spearman’s rho = −0.24, *P* = 0.001), GNRI (Spearman’s rho = −0.23, *P* = 0.001), and serum albumin levels (Spearman’s rho = −0.16, *P* = 0.03).

### Association between chemerin levels and outcomes

**Figure 1:**
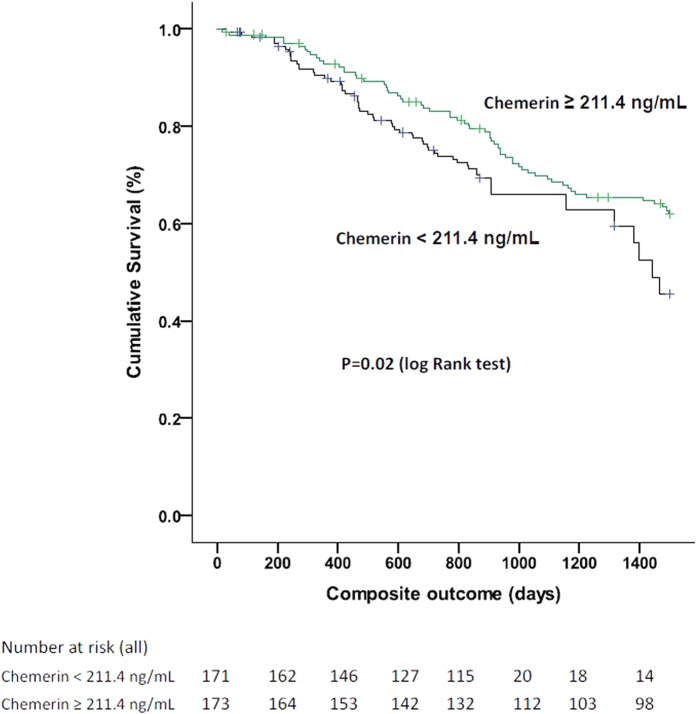
Kaplan-Meier cardiovascular composite outcome analysis for chemerin in prevalent hemodialysis patients.

In the multivariate Cox regression model, patients with higher chemerin levels (≥211.4 ng/mL) had a lower incidence of CV events (adjusted hazard ratio [HR], 0.64; 95% confidence interval [CI], 0.41–0.98) and of composite CV outcomes (adjusted HR, 0.67; 95% CI, 0.45–0.89) compared with patients with lower chemerin levels (<211.4 ng/mL; Fig. 1 and Table 2). After including death as a competing risk of CV events and composite CV outcomes, patients with higher chemerin levels still demonstrated a lower incidence of CV events (adjusted HR, 0.7; 95% CI, 0.46–0.89) and composite CV outcomes (adjusted HR, 0.7; 95% CI, 0.46–0.89) than those with lower chemerin levels did (Table 2). Because chemerin levels and GNRI were positively correlated with each other, we observed an interaction between chemerin levels and GNRI in predicting composite CV outcomes in the Cox regression model. The predictive power of chemerin on CV outcomes was amplified after adjusting for the chemerin × GNRI interaction term; however, chemerin levels still independently predicted CV outcomes in different statistical conditions (Table 2).

### Interaction of adiponectin levels in the relationship between chemerin levels and outcomes

Given that chemerin and adiponectin levels were negatively correlated with each other, we elucidated a non-significant potential interaction between chemerin and adiponectin levels in the prediction of CV outcomes (*P* = 0.07, for interaction). As shown in Table 3, in the fully adjusted model, patients with higher chemerin levels still had more favourable CV outcomes after adjusting for adiponectin levels, and their HRs of CV outcomes further declined after including death as a competing risk. Prevalent HD patients with higher adiponectin levels had a higher risk of composite CV outcomes (adjusted HR, 1.1; 95% CI, 1.0–1.4, per each 10.0 μg/mL increase in adiponectin). Nevertheless, its predictive powers in CV outcomes were no longer significant after including death as a competing risk (Table 3).

## Discussion

The main findings of this prospective cohort study are that prevalent HD patients with higher chemerin levels experience fewer CV events as per our Cox regression or competing risk model. Furthermore, HD patients with higher adiponectin levels tended to have more CV events. Finally, HD patients with higher chemerin levels had a higher nutrition status but lower adiponectin levels. To the best of our knowledge, this is the first investigation to identify the aforementioned associations and demonstrate the protective effects of chemerin on CV outcomes in prevalent HD patients with a median HD vintage of approximately 4.8 years before study entry and negligible residual renal function.

Serum chemerin levels are independently correlated with the GFR and these levels elevate as GFR declines^9^. Therefore, the chemerin levels of HD patients with a long HD vintage and negligible GFR in our cohort were higher than those reported by a previous study enrolling incident HD patients with significant residual GFR. In brief, incident and prevalent HD patients form two patient groups with variable residual GFR, and therefore, a different clinical relevance of chemerin can be anticipated in both these patient groups.

Accumulating evidence has shown that HD patients with high nutritional status have superior CV outcomes^17^,^18^,^19^. Our results showed a novel positive association of chemerin levels with GNRI and serum albumin levels in HD patients. In their animal study, Stelmanska *et al*. demonstrated an approximately two-fold decrease in chemerin mRNA expression in rat white adipose tissue after manipulating the rats with prolonged food restriction; however, after the rats began re-feeding, a four-fold increase was observed in chemerin mRNA expression^20^. Similarly, obese people have higher serum chemerin levels, which decline substantially after bariatric surgery^1^. These findings support the association between chemerin levels and nutritional status. Regarding the findings that malnutrition leads to worse CV outcomes and death in prevalent HD patients^13^,^21^, we believed that prevalent HD patients with higher chemerin levels would have a superior nutritional condition and consequently more favourable CV outcomes. Second, in our previous work, focusing on prevalent HD with fatty liver, we revealed that HD patients with higher chemerin levels have higher fetuin A levels^10^. Both chemerin and fetuin A are secreted by the liver, and their gene expression is amplified in patients with a fatty liver^22^,^23^. Crucially, HD patients with higher fetuin A levels have substantially superior CV outcomes^11^,^12^,^24^. Therefore, the positive association between chemerin and fetuin A levels in HD patients potentially leads to the protective effects of chemerin on CV outcomes. Third, visceral adipose tissue is a detrimental factor in CV outcomes in HD patients^25^,^26^,^27^. The results of the Framingham Heart Study indicated that visceral adipose tissue is a stronger risk factor for a metabolic risk profile than subcutaneous adipose tissue is^28^, which is the case for CV outcomes in HD patients as well^29^. Notably, in their crucial basic study, Goralski *et al*. clearly demonstrated that chemerin is mainly expressed in subcutaneous adipose tissue^30^. Therefore, chemerin is mainly secreted from relatively ‘protective’ subcutaneous adipose tissue and might be associated with superior or at least neutral effects on CV outcomes. However, the respective contribution of subcutaneous and visceral fat to chemerin levels is difficult to assess, particularly in HD patients.

To further clarify the effects of chemerin on CV outcomes, we adjusted the results for adiponectin levels, another major adipokine leading to metabolic disorder and CV disease, in our analysis (Table 3). We observed that the protective effect of chemerin on CV outcomes was further strengthened (HR, 0.48 for CV events and 0.56 for composite CV outcomes; Table 3) after adjusting for adiponectin levels. The consistency of this result was robust when we included death as a competing risk of CV outcomes in the analysis. Notably, the effects of adiponectin levels on CV outcomes in HD patients are inconclusive^14^,^15^. However, recent studies have reported that higher adiponectin levels appear to increase the risks of sudden cardiac death, stroke^14^,^15^ and all-cause mortality^16^. These studies reported findings similar to our observations (Table 3) and indicated that higher adiponectin levels are correlated with higher CV and all-cause mortality in prevalent HD patients.

Collectively, the negative correlation between chemerin and adiponectin may partially contribute to the superiority of CV outcomes in our HD patients. Nevertheless, the knowledge regarding the pathophysiology of adiposopathy in HD patients is extremely limited. Furthermore, a 2016 review article^31^ concluded that the contribution of chemerin to renal disease-associated complications in CKD patients requires further elucidation. Moreover, the mechanisms of the association of adipokines with endocrine and immune disorders contributing to metabolic disease and CV morbidity require further extensive research.

The strengths of this study are its prospective design, optimal observational period, full adjustment for multiple CV outcome-associated factors in HD patients as well as for adiponectin in the outcome analysis, and sensitivity analysis considering death as a competing risk of CV outcomes. However, this study has some limitations. First, we used cross-sectional data to presume longitudinal relationships. The chemerin and adiponectin data were based on the measurements at study entry; however, obtaining serial adipokine data and performing time-dependant analyses to further understand the effect of chemerin on CV outcomes would be more precise. Moreover, the observational design of our study precludes the conclusions of a causal relationship. Second, this was a single-centre study, and all participants were Taiwanese; therefore, our conclusions cannot be generalised to other ethnicities. Third, we did not evaluate traditional anthropometric variables, such as subjective global assessment, handgrip strength and actual lean body mass, to evaluate the nutritional status of prevalent HD patients. However, the nutritional index that we adopted, i.e. the GNRI, is a validated precise and simple index for nutritional assessment in HD patients^32^. Therefore, the nutritional assessment in our study is reliable. Fourth, we did not assess the different adiponectin isoforms and analyzed only the most biologically active form, the high molecular weight isoform adiponectin.

In summary, our results suggest that prevalent HD patients with negligible GFR with higher chemerin levels experience fewer CV outcomes, regardless of whether death is included as a competing risk. Our findings remained robust after adjusting for adiponectin levels. In addition, chemerin levels were positively correlated with nutritional index and negatively with adiponectin levels in our patients. Longitudinal changes in chemerin and their effect on outcomes, mechanisms of adiposopathy between chemerin and adiponectin, and distribution of body adipose tissue in prevalent HD patients warrant future detailed studies.

## Methods

### Patients

This was a prospective study performed using two pooled patient cohorts. The first cohort was composed of 216 prevalent HD patients, whereas the second contained 220 prevalent HD patients. These patient cohorts have been described in more detail previously^10^,^24^. In brief, the two cohorts were collected prospectively to investigate the associations between fetuin A, chemerin, adiponectin, and inflammatory markers with specific outcomes (such as CV events) in prevalent HD patients at Far Eastern Memorial Hospital (FEMH) from 2010 to 2012. The exclusion criteria of the two cohorts were as follows: (1) active infection, (2) recent hospitalisation within 3 months, (3) psychotic illness or other communication problem; (4) active malignancy, (5) aged younger than 20 years, (6) receiving HD for less than 3 months, and (7) patient refusal. In the flow diagram (Supplement Fig. 1), we have clearly shown the enrolment process and the reasons for exclusion in this cohort study. All patients provided written informed consent, and the ethics committee of FEMH approved the study protocol. All experiments were performed in accordance with the relevant guidelines and regulations (FEMH-IRB-100103-E; FEMH-IRB-102132-E; ClinicalTrials.gov; NCT01457625).

In total, 343 HD patients (age, 59 ± 12 years; 171 women) at the Far Eastern Memorial Hospital, Taiwan, were enrolled from February 2010 (first cohort) and March 2012 (second cohort). The enrolled HD patients underwent 3.5–5 h of HD with a bicarbonate dialysate, three times a week. The median HD vintage before recruitment was 4.8 years (range, 0.4–25.9 years), and 93% enrolled patients had anuria.

### Measurement of serum chemerin levels

Serum chemerin levels were determined by using a commercial enzyme-linked immunosorbent assay kit from R&D Systems, Inc. (Minneapolis, MN, USA). The intra- and inter-assay coefficients of variation were 4.5% and 7.9%, respectively. The minimum detectable dose (MDD) of chemerin was 1.08–7.80 pg/mL. The mean MDD was 4.13 pg/mL (SD, 1.03 pg/mL). The blood samples used to measure chemerin levels were obtained after recruitment and were immediately centrifuged and stored at −70 °C until use.

### Measurement of serum adiponectin levels

Serum adiponectin levels were determined using a commercial enzyme-linked immunosorbent assay kit from AssayPro (St. Charles, MO, USA). The intra- and inter-assay coefficients of variation were 3.0% and 8.3%, respectively. The MDD of adiponectin was 0.781–50 ng/mL. The blood samples used to measure adiponectin levels were obtained after recruitment and were immediately centrifuged and stored at −70 °C until use.

### Measurement of clinical parameters and nutritional status

Patient demographic data, concurrent CV disease history, waist circumference (WC), and smoking status were recorded. Venous blood was sampled after fasting for more than 8 hours before the HD patient’s mid-week HD session. Whole blood samples were obtained for haemoglobin measurement, whereas serum was used for determining creatinine, calcium, phosphorus, potassium, uric acid, albumin, and lipid profiles. Intact parathyroid hormone levels were determined using immunoassay (Roche Modular E170 Analyzer, Roche Diagnostics GmbH, Mannheim, Germany). hs-CRP levels were determined using an immunonephelometric method with a Tina-quant CRP (Latex) ultrasensitive assay (D & P Modular Analyzer, Roche Diagnostics GmbH). Estimated GFRs were measured using the Modification of Diet in Renal Disease Study equation as follows: GFR (in mL/min/1.73 m^2^) = 175 × (Scr, serum creatinine, in mg/dL)^−1.154^ × (Age, in years)^−0.203^ × (0.742, if female).

The nutritional status of the participants was quantified using the GNRI, calculated on the basis of serum albumin levels and body weight as follows: GNRI = [14.89 × albumin level (in g/dL)] + [41.7 × body weight/WLo], where WLo is the ideal body weight calculated in the Lorentz equation. The GNRI has been validated in HD patients, and a higher GNRI indicates high nutritional status^32^.

### Outcomes

The primary outcomes were CV events and composite CV outcomes (CV events and CV mortality). CV events were defined as the new occurrence of a CV event, including coronary events (non-fatal myocardial infarction, unstable angina, and coronary revascularization), hospitalised heart failure, hospitalised incident stroke (either ischemic or hemorrhagic stroke), and incident peripheral arterial occlusive disease requiring surgery. CV mortality was defined as death caused during the aforementioned CV events. Analyses of CV events and composite outcomes were performed while accounting for the competing risk of death as well. CV mortality was not included in the primary outcome analysis due to the low incidence of CV death during follow-up (<3% per year in this cohort). The outcome information was centrally adjudicated, in accordance with pre-specified definitions, by trained clinicians and nephrologists.

Follow-up began in February 2010 (first cohort) or March 2012 (second cohort) and was censored on the date of the first CV event, at the end of the study (August 1, 2015), on the date of death or renal transplantation, or when patients were transferred to other HD facilities and were no longer followed, whichever occurred first.

### Statistical analysis

Continuous data are presented as the mean ± SD or median (interquartile range), and categorical data are reported as percentages. The correlations among chemerin, adiponectin, and hs-CRP levels and GNRI were tested using the Spearman’s correlation. Because chemerin levels were not normally distributed in the HD patients (*P* < 0.001 by the Kolmogorov–Smirnov or Shapiro–Wilktest), we constructed plots of chemerin levels and HRs of the composite outcome by using the ‘lowess’ function. The results revealed a non-linear relationship; hence, we stratified the patients into two groups according to their chemerin levels in the outcome analysis. Differences in the baseline characteristics and biochemical parameters between subjects with higher and lower chemerin levels were compared using Student’s *t* and Mann–Whitney *U* tests. The chi-squared test was used for categorical variables.

The outcome analysis was performed using Cox proportional hazard models. We used the ‘Enter’ method to analyse the HR of each primary predictor variable in the model. The primary predictor variable was chemerin levels (higher or lower than the median level of 211.4 ng/mL). Given the pathophysiological links among GNRI and chemerin and adiponectin levels, we further tested the association among them and the potential interaction of these three factors for predicting CV outcomes: we first adjusted for sex, age, HD vintage, DM status, hypertension history, patient cohort, haemoglobin level, WC, GNRI, intact parathyroid hormone (iPTH), calcium phosphate product level, and hs-CRP level; we further adjusted for adiponectin levels in the fully adjusted model. Sensitivity analyses were performed to test the robustness of our findings. Analyses of CV events or composite outcomes were performed while accounting for the competing risk of death using the method of Fine and Grey^33^ because death may be an informative censoring event. All statistical analyses were performed using SPSS software (version 19.0; SPSS, Inc., Chicago, IL, USA) and Stata IC (version 14;StataCorp, College Station, TX, USA). A *P* value of <0.05 was considered statistically significant.

## Additional Information

**How to cite this article**: Chen, H.-Y. *et al*. Reappraisal of effects of serum chemerin and adiponectin levels and nutritional status on cardiovascular outcomes in prevalent hemodialysis patients. *Sci. Rep.* **6**, 34128; doi: 10.1038/srep34128 (2016).

## Supplementary Material

Supplementary Information

## Figures and Tables

**Table 1:** Baseline characteristics of all patients and the patients with low and high chemerin levels.

	All patients	Low chemerin level	High chemerin level	P value
	n = 343	n = 172	n = 171	
Age (years)	59 ± 12	58 ± 12	60 ± 12	0.5
Female sex (%)	50	50	49	0.9
Diabetes mellitus (%)	43	47	40	0.2
Dialysis vintage (years)	5.9 ± 4.9	6.1 ± 5.3	5.6 ± 4.5	0.3
History of hypertension (%)	73	72	75	0.3
History of previous CVD (%)	24	28	20	0.06
Systolic BP (mmHg)	146 ± 31	146 ± 44	147 ± 70	0.1
Diastolic BP (mmHg)	84 ± 13	86 ± 19	83 ± 14	0.2
BMI (kg/m^2^)	23.1 ± 3.7	23.3 ± 3.7	22.8 ± 3.7	0.3
Waist circumference (cm)	86 ± 11	87 ± 11	85 ± 10	0.1
Laboratory data				
Hemoglobin (g/dL)	11.0 ± 1.4	10.9 ± 1.3	11.2 ± 1.4	0.08
Cre (mg/dL)	10.9 (9.6, 12.5)	10.7 (9.4, 12.4)	11.1 (9.7, 12.5)	0.3
eGFR (MDRD, ml/min/1.73 m^2^)	4.4 (3.9, 5.2)	4.5 (3.9, 5.2)	4.4 (3.9, 5.0)	0.4
K (mmol/L)	4.5 ± 0.7	4.5 ± 0.7	4.6 ± 0.7	0.2
Ca (mg/dL); corrected	9.4 (9.0, 9.8)	9.3 (8.9, 9.7)	9.5 (9.1, 9.9)	0.01
P (mg/dL)	5.0 (4.3, 5.9)	5.1 (4.4, 5.9)	4.9 (4.2, 5.9)	0.2
CaxP	47 (39–57)	47 (40, 57)	47 (38, 57)	0.6
iPTH (pg/mL)	260 (122–575)	288 (136, 552)	238 (96, 649)	0.5
hs-CRP (mg/L)	2.9 (1.3–7.5)	3.2 (1.2, 8.0)	2.5 (1.3, 6.3)	0.3
Albumin (g/L)	4.0 ± 0.3	4.0 ± 0.4	4.1 ± 0.3	0.05
T-CHO (mg/dL)	176 (148, 203)	178 (145, 207)	174 (150, 200)	0.8
TG (mg/dL)	127 (81, 198)	119 (75, 190)	132 (89, 205)	0.06
GNRI	103.5 (98.5, 109.0)	103.3 (98, 108.7)	105.8 (99.7, 112.2)	0.05
Chemerin (ng/mL)	211.4 (173.0, 241.9)	173.0 (137.1, 189.1)	215.7 (179.2, 250.6)	<0.001
Adiponectin (ug/mL)	11.5 (7.5, 17.4)	14.4 (10.2, 20.3)	10.8 (7.3, 16.4)	0.015
Medications (%)				
ESA	91	91	90	0.3
HMG-CoA reductase inhibitors	21	23	20	0.3
Anti-hypertensive agents	57	58	55	0.3

Abbreviations: CVD, cardiovascular disease; BP, blood pressure; BMI, body mass index; Cre, creatinine; eGFR, estimated glomerular filtration rate; MDRD, Modification of Diet in Renal Disease; CaxP, calcium phosphate product; iPTH, intact parathyroid hormone; hs-CRP, high-sensitive C-reactive protein; T-CHO, total cholesterol; TG, triglyceride; GNRI, geriatric nutritional risk index; ESA, erythropoiesis-stimulating agents.

*Note:* Conversion factors for units: hemoglobin in g/dL to g/L, ×10; serum calcium in mg/dL to mmol/L, ×0.2495; serum phosphate in mg/dL to mmol/L, ×0.3229; serum T-CHO in mg/dL to mmol/L, ×0.02586; serum TG in mg/dL to mmol/L, ×0.01129; serum albumin in g/dL to g/L, ×10. No conversion is necessary for serum iPTH in pg/mL and ng/L; serum potassium in mEq/L and mmol/L.

**Table 2:** Hazard ratios (HR) of chemerin levels in predicting the outcomes using Cox proportional hazards regression models with multivariate adjustments.

Variables	CV event	Composite CV outcome	CV event (death as competing risk)	Composite CV outcome (death as competing risk)
	Adjusted HR (95% CI)	Adjusted HR (95% CI)	Adjusted HR (95% CI)	Adjusted HR (95% CI)
Chemerin (higher v.s lower)	0.64 (0.41‒0.98)§	0.67 (0.45‒0.89)§	0.7 (0.46‒0.89)§	0.7 (0.46‒0.89)§
GNRI (every 1 unit increase)	0.93 (0.89‒0.98)§	0.92 (0.82‒0.98)§	0.9 (0.79‒0.97)§	0.88 (0.75‒0.97)§
Chemerin (adjust for chemerin x GNRI)				
(higher v.s lower)	0.38 (0.19‒0.75)ǂ	0.54 (0.2‒0.8)ǂ	0.52 (0.24‒0.78)ǂ	0.58 (0.19‒0.83)ǂ
Interaction	0.04	0.007	0.02	0.004

Abbreviations: CV, cardiovascular; HR, hazard ratio; CI, confidence interval; GNRI, geriatric nutritional risk index.

^§^Adjusted for gender, age; dialysis vintage; presence of diabetes mellitus, patient cohort, waist circumference, geriatric nutritional risk index (GNRI), calcium phosphate product (CaxP), hemoglobin, intact parathyroid hormone (iPTH) and high-sensitive C-reactive protein (hs-CRP) levels.

^ǂ^Adjusted for gender, age; dialysis vintage; presence of diabetes mellitus, patient cohort, waist circumference, geriatric nutritional risk index (GNRI), chemerin x GNRI, calcium phosphate product (CaxP), hemoglobin, intact parathyroid hormone (iPTH) and high-sensitive C-reactive protein (hs-CRP) levels.

**Table 3:** Hazard ratios (HR) of chemerin and adiponectin in predicting the outcomes using full-adjusted Cox proportional hazards regression models.

Variables	CV event	Composite CV outcome	CV event (death as competing risk)	Composite CV outcome (death as competing risk)
	Adjusted HR (95% CI)§	Adjusted HR (95% CI)§	Adjusted HR (95% CI)§	Adjusted HR (95% CI)§
Chemerin (higher v.s lower)	0.48 (0.26‒0.9)	0.56 (0.31‒0.9)	0.46 (0.22‒0.84)	0.55 (0.31‒0.87)
Adiponectin (per each 10.0-ug/mL increase in adiponectin)	1.0 (0.95‒1.42)	1.1 (1.0‒1.4)	1.1 (0.89‒1.33)	1.09 (0.88‒1.48)

Abbreviations: CV, cardiovascular; HR, hazard ratio; CI, confidence interval.

^§^Adjusted for gender, age; dialysis vintage; presence of diabetes mellitus, patient cohort, waist circumference, geriatric nutritional risk index (GNRI), calcium phosphate product (CaxP), hemoglobin, intact parathyroid hormone (iPTH), high-sensitive C-reactive protein (hs-CRP) and adiponectin levels.
