# Supplementary material for: Reappraisal of effects of serum chemerin and adiponectin levels and nutritional status on cardiovascular outcomes in prevalent hemodialysis patients
Source: Sci Rep. 2016 Sep 26;6:34128. doi: 10.1038/srep34128 (PMC5036174; doi:10.1038/srep34128)

Reappraisal of effects of serum chemerin and adiponectin levels and nutritional status on cardiovascular outcomes in prevalent hemodialysis patients

Hung-Yuan Chen[1,2], MD; Yen-Lin Chiu[1,2], MD, PhD; Shih-Ping Hsu[1,2], MD, PhD; Mei-Fen Pai[1,2], MD; Ju-Yeh Yang[1,2], MD, MPh; Hon-Yen Wu[1,2], MD, PhD; Yu-Sen Peng[1,2,*],MD, PhD

Abstract word count: 176

Word count: 3088

Number of tables: 3

Number of figures: 1

Supplement figure: 1

Running title: Chemerin and cardiovascular outcomes in HD patients

ClinicalTrials.gov: NCT01457625

[1]Division of Nephrology, Department of Internal Medicine, Far Eastern Memorial Hospital, New Taipei City, Taiwan.

[2]Division of Nephrology, Department of Internal Medicine, National Taiwan University Hospital and National Taiwan University College of Medicine, Taipei, Taiwan

Supplement Figure 1. Study flow diagram

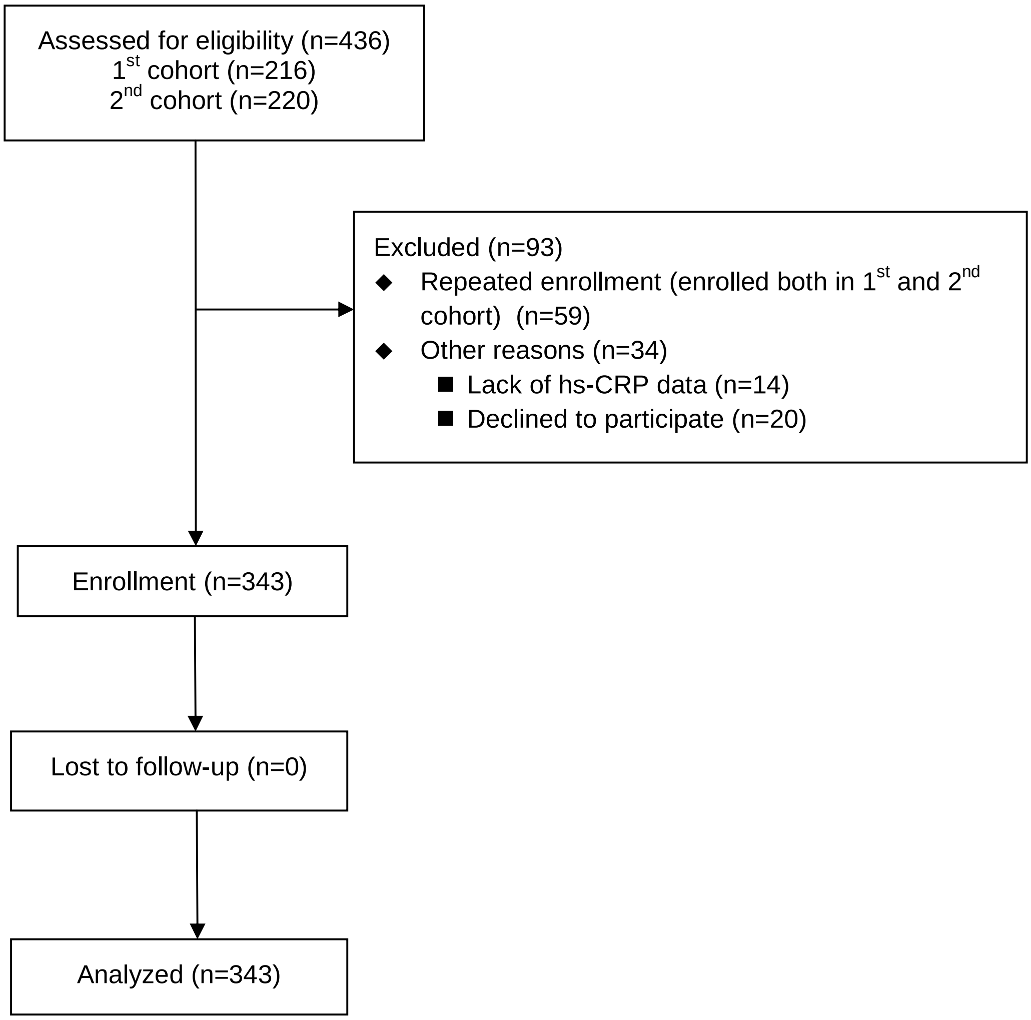

Supplement: Supplementary Information [file srep34128-s1.pdf]
